# UEPG Abraça Program: The importance of a psychosocial care service in the university context

**DOI:** 10.1192/j.eurpsy.2021.2010

**Published:** 2021-08-13

**Authors:** L. Floriano, C. Brabicoski, E. Pinto, L. Monteiro

**Affiliations:** Nursing And Public Health, State University of Ponta Grossa, Ponta Grossa, Brazil

**Keywords:** mental health, Promotion of Health, University

## Abstract

**Introduction:**

In 2018, it was implanted in a Brazilian public university, the UEPG Abraça Extension Program, which has as objectives to realize psychosocial care and accompaniment, offer psychotherapies and therapeutic groups to the university community, that is, students, professors and university staff to suicide prevention, coping with mental disorders and the problematic use of alcohol and other drugs. The Program has a multi-professional team composed of nurses, social worker, psychologists and psychiatrists, in an exclusive environment, thus guaranteeing comfort and secrecy to users.

**Objectives:**

To characterize the socio-demographic and clinical profile of users assisted by the UEPG Abraça Program and to disclose the importance of the service for the promotion of Mental Health.

**Methods:**

Qualitative, quantitative, descriptive study, with a structured questionnaire as the research instrument. The collection took place in 2019 and the target audience were users who sought psychological care.

**Results:**

There were 469 admissions/visits and 35 of them continued with individual visits in 2020. The majority of users were female (58%) and had the age group between 17 and 20 years. The main mental health problems identified were anxiety, self-knowledge and university-related problems such as interpersonal conflicts and difficulties in the work process. The most attended undergraduate courses were Letters, Journalism and Dentistry.

**Conclusions:**

It is noted the importance of a psychosocial care service at university level for the care and promotion of Mental Health.

**Disclosure:**

No significant relationships.

